# Patients’ Representations of Perceived Distance and Proximity to Telehealth in France: Qualitative Study

**DOI:** 10.2196/45702

**Published:** 2024-04-26

**Authors:** Amélie Loriot, Fabrice Larceneux, Valérie Guillard, Jean-Philippe Bertocchio

**Affiliations:** 1 Paris Dauphine–PSL (Paris Sciences & Lettres) University Paris France; 2 Service Thyroïde – Tumeurs Endocrines, Hôpital de la Pitié-Salpêtrière Assistance Publique – Hôpitaux de Paris Paris France; 3 SKEZI Annecy France

**Keywords:** telehealth, teleconsultation, social representations, perceived proximity, semiotic square

## Abstract

**Background:**

In the last 2 decades, new technologies have emerged in health care. The COVID-19 pandemic further accelerated the adoption of technology by both health care professionals and patients. These technologies create remote care practices that bring several benefits to the health care system: easier access to care, improved communication with physicians, and greater continuity of care. However, disparities in the acceptance and use of telehealth tools still exist among patients. These tools also disrupt conventional medical practices and prompt a new reassessment of the perceptions of distance and proximity as physical (ie, time and space dimensions) and nonphysical (ie, behavioral dimensions) concepts. The reasons why patients do or do not adopt telehealth tools for their care and therefore their perspectives on telehealth remain unanswered questions.

**Objective:**

We explored the barriers as well as the motivations for patients to adopt telehealth tools. We specifically focused on the social representations of telehealth to establish a comprehensive conceptual framework to get a better understanding of how telehealth is perceived by patients.

**Methods:**

This study uses a qualitative design through in-depth individual interviews. Participants were recruited using a convenience sampling method with balanced consideration of gender, age, location (urban/rural), and socioeconomic background. After collecting informed consent, interviews were transcribed and analyzed using the thematic analysis methodology.

**Results:**

We conducted 14 interviews, with which data saturation was reached. The 2 main opposed dimensions, perceived proximity and distance, emerged as an essential structure for understanding the social representations of telehealth. A logic of engagement versus hostility emerged as the main tension in adopting telehealth, almost ideological. Interestingly, practical issues emerged regarding the adoption of telehealth: A logic of integration was opposed to a logic of constraints. Altogether, those dimensions enabled us to conceptualize a semiotic square, providing 4 categories with a coherent body of social representations. Due to the dynamic nature of these representations, we proposed 2 “paths” through which adherence to telehealth may improve.

**Conclusions:**

Our semiotic square illustrating patients’ adherence to telehealth differentiates socially beneficial versus socially dangerous considerations and pragmatic from ideological postures. It shows how crucial it is to consider perceived distance and proximity to better understand barriers and motivations to adopting telehealth. These representations can also be considered as leverage that could be modified to encourage the step-by-step adhesion process. Even if reducing the perceived temporal distance to in-person meeting and enhancing the perceived proximity of access to care may be seen as efficient ways to adopt telehealth tools, telehealth can also be perceived as a care practice that threatens the patient-physician relationship. The patient-oriented perceived value turns out to be critical in the future development of and adherence to telehealth tools.

## Introduction

### Telehealth, a Subset of eHealth Still Ongoing

Many technologies have been developed in eHealth in recent years. Defined as the “use of information and communication technologies in support of health and health-related fields, including health care services, health monitoring, health literature, and health education, knowledge and research” [1], eHealth covers a wide range of practices. First, mobile apps and connected devices are referred to as mobile health (mHealth). Second, telehealth (ie, the practice of medicine using information and communication technologies) covers 5 practices: teleconsultation, teleexpertise, medical regulation, remote medical monitoring, and remote medical assistance.

Recent research focusing on remote care has indicated some confusion regarding the wording used to refer to health-related technologies [2]. For instance, the terms “telemedicine” and “telehealth” are often used interchangeably [3]. However, some researchers highlight a difference between these 2 concepts. Whereas telemedicine is limited to remote clinical services, telehealth is broader and refers to remote clinical services as well as remote nonclinical services, like administrative meetings [4]. Thus, telehealth has been defined as “the use of electronic information and telecommunication technologies to support and promote long-distance clinical health care, patient and professional health-related education, public health and health administration” [5].

eHealth is expected to lead to significant changes in the delivery of care and medical practices [6]. Because of (1) disparities in access to health care, (2) the aging population, and (3) budget constraints limiting public policies, the development of eHealth devices can be seen as a solution to the future challenges faced by the health system in many high-income countries [7]. Before the COVID-19 crisis, there were significant disparities in the use of eHealth tools between European countries [8]. A global shift occurred during the pandemic: The use of many eHealth tools became necessary, democratizing their use in terms of communication, monitoring, or care delivery, and the use of technology to provide health services has accelerated [9,10]. Telehealth may now concern everyone.

### Benefits and Barriers of Telehealth

The practice of telehealth presents many benefits for patients, including (1) better access to health care services, in particular in isolated regions like rural areas; (2) improved continuity of care; (3) increased availability of health information [11]; and (4) empowerment of patients [12]. As such, telehealth is supposed to increase efficiency and quality of care [10] and favors patient-centered care by enabling better communication between patients and health care professionals [13].

However, researchers have pointed out that many barriers exist that limit health equity for all patients. Significant disparities remain regarding the access to, adherence to, and use of telehealth tools [14,15]. In particular, little is known about the role of digital health literacy [13,15,16] (ie, “the ability to search for, find, understand and evaluate health information from electronic sources and to apply knowledge acquired to solve a health problem” [17]). Among individuals in rural areas, low levels of education are associated with lower use of digital health tools [18]. Some scholars argue that online interactions are impersonal and dangerous because of the lack of a physical examination [10] and that telehealth may threaten the quality of the relationship between physicians and patients [19].

Studies among health care professionals have also shown a reluctance to adopt these technologies because of a fear of “dehumanization” by virtualizing patients and care [20]. This feeling of dehumanization of care could explain negative attitudes toward telehealth [21].

Patients tend to attribute significant importance to health care professionals’ physical and emotional presence [22] and direct interactions with them [7]. However, the digitalization of health is transforming these relationships [23]: Telehealth disrupts medical practices and reduces physical interactions between patients and physicians. However, it leads to reconsidering notions of distance and proximity [23], including physical and nonphysical dimensions (ie, cognitive or relational aspects that are perceived by individuals) [24-26]. Physical proximity and perceived proximity are not necessarily aligned. Indeed, individuals can feel themselves close to an element that is physically far but also to perceive it far when it is physically close [26]. Perceived proximity has a cognitive dimension that refers to “a mental assessment of how distant someone else seems” and an affective component, since these representations are subject to emotions rather than rational thought [26,27]. In health care, Talbot et al [28] investigated the perceptions French physicians may have about telehealth using the conceptual framework of proximity of Boschma [25] that includes the following 5 dimensions of proximity: cognitive, organizational, social, institutional, and geographical. However, how patients react to these changes in care delivery and the representations of these practices remain unanswered questions. Therefore, exploring patient’s representations of telehealth is important to better understand psychological mechanisms underlying the adherence to telehealth. The theory of social representations is fruitful in overcoming this limitation.

### The Social Representations Theory

The theoretical background of social representations provides a framework for understanding how new concepts become common knowledge. Defined as a collective elaboration “of a social object by the community for the purpose of behaving and communicating” [29], social representations consist of a system of values, ideas, and practices that enable individuals to orient themselves in their material and social world as well as to master it and provide a code for social exchange [30]. Therefore, social representations provide people with a common frame of communication that is built in everyday interactions. More precisely, a social representation corresponds to thoughts and feelings being expressed in verbal and overt behavior of actors that constitutes an object for a social group [31].

Although social representations are commonly shared, some may be more polemical, reflecting oppositions between social groups in society [32]. In addition, social representations have a dynamic nature across and within social groups of people, and societal practices, communication, and the process of knowledge are strongly connected, particularly in the health field, which has been one of the leading research areas for this theory [33-35].

Interestingly, social representations constitute a structure explaining behaviors that result not only from an individual cognitive process but also from social and cultural representations and that are shared collectively [36,37]. Social representations have been shown to be a significant indicator of attitudes [38,39]. However, social representations of patients have never been studied in the context of telehealth specifically. A qualitative study is well suited to understand these representations. The objective of our qualitative research was to establish a comprehensive conceptual framework to gain a better understanding of how telehealth influences perceived proximity or distance for patients and therefore, to better apprehend their barriers as well as their motivations to adopting telehealth tools.

## Methods

### Study Design

A qualitative study was conducted with an interpretative approach to explore patients’ representations of telehealth and their perception of proximity toward it. We adopted an inductive, constructivist perspective, assuming that people construct their life-worlds through their representations and interpretations of telehealth as a social fact to which they attribute specific terms and meanings.

### Setting and Sample

Qualitative in-depth individual interviews were set up using a semistructured thematic interview guide. Convenience sampling was used to recruit participants. Variation sampling was sought [40] with consideration of gender, age, location (urban/rural), and socioeconomic background (Multimedia Appendix 1). We used the saturation criterion to stop recruitment. This criterion is the point at which gathering more data about a theoretical construct reveals no new properties nor yields any further theoretical insights [41]. This saturation point is usually reached with 9 to 17 interviews [42].

### Data Collection

After obtaining informed consent, patients were contacted, and an appointment for an interview was set. Interviews lasted from 45 minutes to 75 minutes and were performed directly inside the family home or conducted through the digital platform Microsoft Teams because of the geographical distance between the researcher and the participant. The study took place in May 2022. A total of 14 interviews were gathered: 8 participants were female, 6 were male, and their mean age was 52 (range 23-83) years. Of the interviews, 11 interviews were run face to face, and 4 were online.

The interview guide explored various aspects of how health and telehealth are perceived; including defining what constitutes perceived good health; understanding respondents’ relationship with their own health; examining how they seek health-related information; discussing challenges in accessing care as related to geographical, temporal, and perceived distances; and evaluating respondents’ overall and specific relationships with technology within the context of health care. This comprehensive approach aimed to gain insights into how individuals perceive telehealth and their level of engagement with it.

During each interview, we wrote down our impressions that could possibly impact the interpretation of results. Interviews were digitally audio-recorded with permission, and verbatims were transcribed.

### Ethical Considerations

At the beginning of each interview, potential participants were given comprehensive information about the context, objectives, and methods of the study. The interviewees were informed that they could withdraw from this study at any time. After allowing enough time for any questions or clarification they may have required, all the participants gave their informed consent. The study design was reviewed and approved by the Research Ethics Committee of Paris Dauphine–PSL (Paris Sciences & Lettres) University (20231128/01). Additionally, following national legislation, data were pseudonymized during the transcription process in a way that no participant could be directly identified: A number was assigned to each participant with no record of any directly identifying data. Participants received no compensation for participating in this research.

### Data Analysis

First, we conducted a vertical analysis and read the transcripts to get an impression of the whole data set. Second, transcribed data were analyzed using a horizontal thematic analysis to develop a narrative of the findings through a categorical approach using qualitative software (NVivo Version 12). We followed the grounded theory approach to code verbatim [43]: Each transcript was coded inductively by manually marking central key words that could represent a code. The codes were then grouped under themes that emerged through the analysis process. Finally, we categorized the data by collapsing codes that conveyed similar meanings. Multimedia Appendix 2 presents an example of our analysis process.

After the first step of the analysis of social representations, which was to record all the dimensions that emerged from the participants, we used the semiotic square method to map semantic categories highlighting opposing and complementary concepts [44]. This structure enables the understanding of the tension among symbolic meanings and the elements by which meaning is being expressed [45]. The semiotic square has been often applied in consumer research [46] and specifically to explore consumers’ relationships with technology ideology [47].

## Results

### Overview

First, a specific definition of telehealth emerged from the patient perspective. If researchers define telehealth broadly, the interview analysis revealed that telehealth is associated with teleconsultation for a large majority of patients and rarely with other practices. It concerns mainly remote care and is associated with questions about the quality of interactions with the physician.

Second, the content analysis revealed 4 main types of social representations of telehealth: the expected opposition between engagement and hostility and a more subtle distinction between integration and constraint.

### Representations of Proximity: the Logic of Engagement

Our analysis of interviews revealed the first category of very positive social representations related to telehealth that led to a logic of engagement and adherence to this practice. This commitment is based on the idea of optimization of health services. The strong proximity with its practice is explained by a feeling of comfort and a perception of convenience. Telehealth is considered an easy, practical tool. Participant 4 (P4) mentioned:

I found it practical and comfortable.

Perceived practicality and convenience underline the actual benefits of adopting telehealth. Indeed, this practice enables a reduction of the perceived temporal distance to the consultation, leading to representations of efficiency and effectiveness (P13) on one hand and allowing reinforcement of access to care, which creates a feeling of personal usefulness (P8), on the other hand. Participant 13 (P13) mentioned:

Now that everything is overbooked in their appointments, (...), we are at about 15 days/3 weeks for getting any new appointment, both by phone or by Doctolib, in video, it is a little faster.

In addition, participant 8 (P8) said:

It is so quick, it makes everyday life easier!

From this perspective, the main issue behind social representations of proximity is related to an improvement of the functional proximity to telehealth.

### Representations of Distance: the Logic of Hostility

At the opposite end to that of the first category, the second category of social representations follows a logic of hostility toward telehealth. It reveals a strong rejection of its development. Although adherence follows a view of functional proximity, rejection is explained by a lack of perceived relational proximity caused by telehealth. These representations of perceived distance reveal a profound fear of the dehumanization of medicine. Telehealth is seen as a dehumanizing practice that is destructive of human interaction by virtualizing both patients and care, as Kaplan [20] mentioned. This was confirmed by participant 6 (P6), who stated:

It kills the human contact, which is really important to me. I definitely prefer having the secretary over the phone to tell me there is an appointment in three weeks.

The major component of this category is the perceived deterioration of the relationship with the physician. Great importance is given to the human dimension in care. However, the interviews revealed these representations are based on a feeling of detachment from the caregiver caused by telehealth. This emphasizes the impersonal nature of the relationship. Participant 2 (P2) stated:

We dematerialize everything. It brings detachment from the caregiver.

From this perspective, the development of a relationship with perceived proximity and trust seems incompatible with distant and remote care. The virtual nature of this link is intrinsically considered as the opposite of human interaction. Participant 1 (P1) stated:

I do not like it. I like to see the person right in front of me.

Here, social representations of telehealth found an increase of perceived distance between the patient and physician. The perception of actual proximity to the physician tends to disappear with telehealth, which reinforces emotional and affective distance [48]. These representations finally highlight the fact that telehealth cannot replace an in-person consultation. For instance, participant 5 (P5) stated:

I would not make [a remote physician] my referring physician. There need to be a close relationship with him. I must be able to give him my trust. I am not sure that I will always have the same doctor when using teleconsultation.

Altogether, these depictions of distance nurture the perception that telehealth has a detrimental or potentially harmful impact on society, as it undermines the interpersonal nature of care.

In addition to these 2 opposite categories of representations, proximity versus distance, more nuanced types of social representations also emerged within the verbatims. We labelled them “nondistance” and “nonproximity” representations.

### Representations of Nondistance: the Logic of Integration

The third category of social representations reflects “nondistance” to telehealth, as these representations are related neither to total adherence nor to rejection but rather follow a logic of integration: Participants highlighted the actual possibility to choose to use (or not) telehealth tools. Representations do not reflect a full engagement with this practice but rather a nonrejection of telehealth.

First, these representations of nondistance highlight the functional aspects of this practice. In this context, developing a relational proximity with the physician was not judged as necessary. For instance, participant 4 (P4) stated:

I felt more like I was with a teleoperator than a physician. It felt like there was a script behind it, but why not, that is not necessarily a bad thing.

This situation is not seen as a problem; the efficient and nonrelational aspect of the consultation is valued here. Thus, this representation shows a greater emphasis on the functional proximity rather than on the relational proximity [24].

The importance given to the functional aspects of telehealth was also revealed through the way specific health practices are elicited. For instance, telehealth was mainly seen as a backup or emergency solution, leading to occasional use according to the situation. Participant 5 (P5) stated:

It can be a first step to detect an emergency. For example, if you cannot get a doctor during the weekend, we have remote visits (...) So, to me, it is an emergency solution.

Because it is convenient, patients do not reject telehealth, especially when there is no need to be seen in person, for example for a prescription renewal, as suggested by participant 4 (P4):

It depends on what you are looking for in the consultation. If it is for a medication renewal, yes, I would recommend it.

Thus, these representations of nondistance do not refer to hostility nor engagement toward telehealth but rather to tolerance. The practice is adopted but not entirely accepted. Indeed, the use of telehealth should remain occasional. Participant 11 (P11) said:

If I were starting using a teleconsultation system, I would say to myself ‘no more than three times in a row.’ The fourth time, you still have to go, once every two years for a check-up, I would tend to say that.

Tolerance comes also with some reluctance about the reliability of this practice. Telehealth was perceived as less reliable than a physical consultation because there is no physical contact and no auscultation, which seems to lead to mistrust, as suggested by participants 10 (P10) and 14 (P14).

Auscultation is one of the first things you learn in medicine, like touching the patient. Try to get an auscultation from a machine, to put its hands on the belly.

P10

When I had my operation, I had a consultation with the anesthesiologist by teleconsultation. It was silly, he told me to pull my tongue out (...) No, for me this is ridiculous!

P14

Overall, social representations related to nondistance reveal nonrejection of telehealth under conditions of efficiency and reliability. The choice of using telehealth tools is made under specific circumstances and leads to occasional use, based on high value placed on simplicity and functional aspects.

### Representations of Nonproximity: the Logic of Constraint

Within the fourth category, social representations are related to “nonproximity,” a label that reflects a logic of constraint. Whereas representations of proximity highlight engagement and active behavior toward telehealth, representations of nonproximity depict situations of the use of telehealth when there is no other choice, as participant 10 (P10) mentioned:

Is telehealth a good thing? Like everyone else, I use it because I am left with no alternative option.

Patients come to telehealth whenever they have no or few alternatives, considering telehealth as a last option, such as during a lockdown for example, as explained by participant 3 (P3):

If I had to use it, it would really be out of obligation, like during a lockdown, and because I do not have the possibility to move around.

In this perspective, telehealth tools are not really accepted and should remain a second option to physical in-person consultations, mainly because telehealth requires digital literacy. Participant 7 (P7) explained:

For the elderly, it is a problem! I have to schedule their appointments from my own mobile phone because they do not have access to the internet.

Thus, like the representations of perceived distance, the representations of nonproximity are also mostly negative. However, they do not reflect a total rejection of the practice of telehealth but rather a nonadherence as patients come to it when they have no other option.

**Figure 1:**
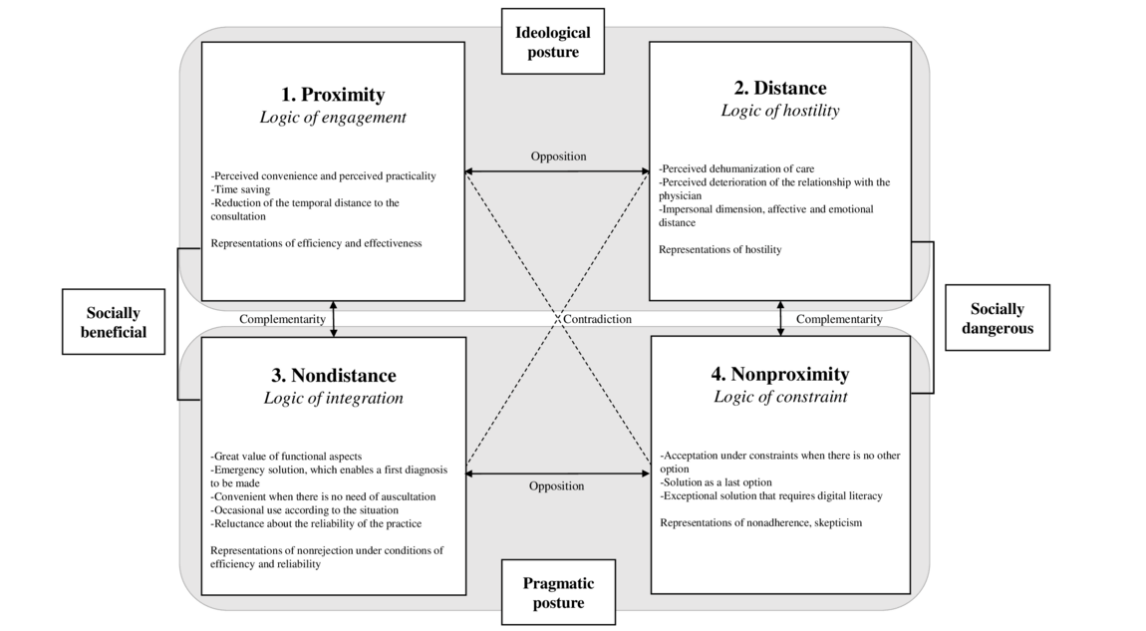
Overview of the 4 categories of social representations of telehealth, including the corresponding logic and main drivers, that resulted from 14 qualitative interviews run with patients in May 2022, with a major horizontal dimension (proximity versus distance) and its opposites (vertical).

Finally, our qualitative analysis allowed us to structure a semiotic square (Figure 1) with 2 main categories of social representations of telehealth (ie, perceived proximity and perceived distance) as well as subsequent tensions in the discourse. The negation of these 2 terms forms 2 other categories illustrating 4 distinctive classes of meanings highlighting nuanced representations of perceived proximity and distance to telehealth and the opposite and complementary relationships [49]. The main components of the 4 categories are summarized in Figure 1. Interestingly, 2 additional analyses of the semiotic square improve our vision of social representations of telehealth, one based on a vertical reading and the other based on a horizontal reading.

### Telehealth: Socially Beneficial Versus Socially Dangerous

From a vertical reading of the relationships between the categories, there are 2 structuring representations of telehealth. In the left part of the semiotic square (Figure 1), the complementary relationship, linking proximity and nondistance, refers to favorable representations as well as to discourse encouraging the development of telehealth. These tools are perceived as socially beneficial for all stakeholders, but there is room for improvement in generalizing their use.

Within these favorable representations of telehealth, adherence and nonrejection are based on 2 main drivers. First, trust in the physician is crucial as he or she is considered a legitimate expert, as suggested by participant 4 (P4):

I feel that doctors are experts (...), I trust them entirely because to me they seem to be experts.

Consequently, positive representations of telehealth seem to be linked to the perceived relational proximity with the health care professional. Second, these representations stem from familiarity with the tool. Being familiar with the term “telehealth” and knowing what it means generate a feeling of closeness toward it. Participant 5 (P5) stated:

I heard [about telehealth] because in my profession—I work with pharmacies—we talk about it.

In the right part of the square is the complementary relationship combining perceived distance and nonproximity. This underlines representations of hostility and skepticism toward telehealth, which are considered socially harmful or even dangerous for society. Rejection and nonadherence seem to be explained mainly by insufficient digital literacy as well as difficulties accessing the Internet and telehealth tools, as suggested by participant 9 (P9):

No, I do not use the Internet at all! (...) There are surely many things to do but I do not know how to do them...

This revealed a substantial cognitive distance to telehealth and ultimately making care practices feel more complex. The ancestral role of auscultation in medical consultation and the importance given to touching patients are noted, showing that the lack of perceived physical proximity between the patient and physician tends to reinforce the psychological distance toward telehealth and ultimately the rejection of its practice. Participant 4 (P4) said:

The ability itself of performing an actual auscultation by touching people and listening to them using a stethoscope is being lost at the expense of the care to improve the development of technology.

### Telehealth: Ideological Versus Pragmatic Postures

A horizontal reading highlights the similarity of the logics of engagement and hostility, both based on ideological postures: pros and cons of the practice of telehealth depending on whether it seems to belong to the “good” versus “bad” for the society. More efficiencies appear to be pros, and less of a human relationship appears to be a con. Conversely, the logics of integration and constrain reflect pragmatic postures: how to deal with the tool and on what occasion. Sometimes, it appears to be accepted because it is convenient and adapted to specific situations, sometimes because there is no other choice. Interestingly, ideological postures tend to separate opposite groups, while the pragmatic views tend to rebuild a link between the nondistance and nonproximity groups. These nuanced, more balanced perceptions invite us to think about the practical implications, elaborating “paths” of social representations to drive patients toward less rejection of and more adherence to telehealth.

## Discussion

### Main Findings

Using qualitative methods, our findings suggest a new conceptual framework to apprehend telehealth from patients’ perspectives based on 4 categories of social representations. First, perceived proximity was associated with social representations reflecting the idea that telehealth is intrinsically an efficient, practical, and effective solution. This logic of engagement is in line with a strong belief in progress and technological tools to face the challenges of the health care system, namely the issue of access to care. On the opposite side, social representations were more related to a feeling of distance from telehealth, enforcing an unfavorable attitude and leading to a rejection of these tools. This logic of hostility is mainly anchored in a fear of dehumanization of society. Telehealth is blamed for compromising the quality of the relations and for accelerating the loss of human contact between patients and physicians. This perceived distance from telehealth highlights a situation of exclusion, especially for patients who do not have access to digital technology or who do not have sufficient digital literacy. Aside from these 2 categories, 2 more nuanced types of representations emerged. First, from a logic of integration, social representations revealed that telehealth is appealing but showed worries and fears about its reliability. This practice can be conditionally accepted according to a situational approach. Second, a logic of constraint reflected social representations based on skepticism but leading to acceptance when there are no alternatives.

From a theoretical point of view, our results, based on a semiotic square, bring new elements to the literature of perceived proximity. We have shown that telehealth leads to reconsidering proximity through several dimensions. Although not diminishing the geographical or physical gap between the patient and the health care provider, technological tools, such as a teleconsultation from home, can enhance accessibility to health care. The relational dimension of proximity, already identified by Boschma [25], seems to also be impacted by telehealth. Indeed, many social representations have shown that this perception of proximity with the caregiver is reduced by telehealth and revealed a fear of dehumanization in the relationship. In addition, we showed that perceived functional proximity to telehealth leads to increased adherence and a favorable attitude to its development, which should encourage policymakers to strengthen this aspect in communication strategies for telehealth. These findings also constitute a societal contribution. In addition, this research has revealed 2 major oppositions embedded in the social representations. The first one consists of “good,” or a socially beneficial position, versus “bad,” or a socially dangerous position. The second one highlights the posture, rather “ideological” or “pragmatic,” leading to contributions to public policy aiming to foster adherence to eHealth tools.

**Figure 2:**
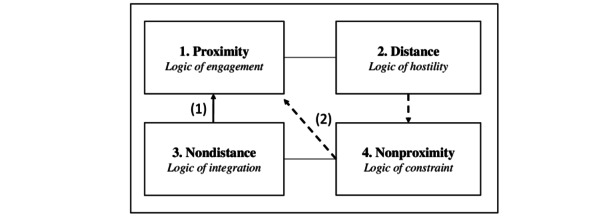
Illustration of 2 paths to create proximity with telehealth valuing either functional (path 1) or relational (path 2) aspects.

Building a semiotic square also revealed potential changes in people’s representations of telehealth and thus the potential to contribute to change attitudes toward these tools. They may be adapted to patients’ concerns and aspects that patients value in the practice of consultation. Our qualitative material brings insight to how these representations can be obstacles to the adoption of telehealth, as well as elements that can foster adherence. We propose considering paths through which patients’ representations could evolve. Mobilizing the social representations along these paths could first alleviate the perception of distance to the health care professional then enable the perceived proximity to telehealth. Our analysis emphasized some risks in how telehealth is implemented. If telehealth is developed without considering representations expressing reluctancy, individuals who are subjected to the use of telehealth may remain hostile to its development, may gradually feel a distance to it, and may finally totally reject this practice (coming from nonproximity to distance). To avoid such a vicious circle, 2 paths (Figure 2) may create an increased feeling of proximity to telehealth.

The first path consists of transforming representations related to a perceived nondistance into a perceived proximity to telehealth. This pathway adopts a functional approach to consultation. The challenge is to dispel fears about the technological feasibility of using digital health tools to eliminate skepticism and reinforce favorable representations. It would then be necessary to reassure patients about the importance of any human contact during medical consultations. Highlighting the regular and immediate exchanges with physicians that telehealth allows would be perceived as helpful. Developing remote auscultation solutions and increasing communication about them by highlighting the accuracy and reliability of these technologies would help to alleviate these concerns and encourage adherence to these tools. Finally, reinforcing the benefits in terms of efficiency, time optimization, and practicality would contribute to (1) reducing the perceived temporal distance of access to care and (2) increasing the perceived functional proximity to telehealth.

The second path consists of (1) transforming representations related to a perceived distance into a feeling of nonproximity and subsequently (2) fostering the perceived proximity to telehealth. This path is mainly aimed at individuals who attach great importance to the relational and human dimension of care. The first challenge would be to strengthen trust in the health care system because representations and attitudes toward telehealth are intrinsically linked to the relationship patients develop with the health care system and physicians. It is also necessary to improve access to digital technology to reduce the cognitive distance and to increase their perception of proximity. Finally, highlighting and communicating about the strengthening of relational and affective proximity, allowed by telehealth when facilitating contact between patients and physicians, could lead to favorable representations and attitudes. Therefore, conceiving a system of medical support with a health care professional in telehealth booths could be an effective solution.

### Limitations and Research Avenues

This study has some limitations. First, our sample did not include patients with a broad range of diseases: Very few of them had chronic diseases. Due to the sample size, we could not cover all medical specialties: For instance, ophthalmology and the need for emergency surgery may bring specific representations of telehealth for patients. It could also be interesting to interview people from other rural areas known as “medical deserts” (ie, regions with inadequate access to health care). In addition, we interviewed patients who do not practice as health care professionals. To broaden our research findings, we could incorporate additional insights by examining the perceptions of telehealth among other groups, particularly caregivers.

### Conclusion

The development of telehealth tools leads to new challenges in medical practice. The social representations telehealth brings go beyond the perception of proximity and distance, are multifaceted, and include postures and attitudes. The social representations revealed by the semiotic square on perceived proximity to telehealth underscore the importance of designing health care strategies based on a patient-centric approach in the implementation of digital health tools.

## Appendix

Multimedia Appendix 1 Characteristics of patients.

Multimedia Appendix 2 Example of the qualitative analysis process used in this research.

## Abbreviations

mHealth: mobile health
PSL: Paris Sciences & Lettres

## Data Availability

The data sets generated and analyzed during this study are available upon reasonable request from the corresponding author.
