# Supplementary material for: Patients’ Representations of Perceived Distance and Proximity to Telehealth in France: Qualitative Study
Source: J Med Internet Res. 2024 Apr 26;26:e45702. doi: 10.2196/45702 (PMC11087856; doi:10.2196/45702)
Supplement: Multimedia Appendix 1 [file jmir_v26i1e45702_app1.docx]

Appendix 1: Characteristics of patients

|  | *Gender, age, location, socioeconomic backgrounds* |
| --- | --- |
| *P1* | *F, 60, rural, +* |
| *P2* | *H, 45, urban, -* |
| *P3* | *H, 23, urban, ++* |
| *P4* | *F, 57, urban, ++* |
| *P5* | *H, 57, urban, ++* |
| *P6* | *H, 62, rural, --* |
| *P7* | *F, 54, rural, -* |
| *P8* | *F, 72, rural, -* |
| *P9* | *H, 83, rural, +* |
| *P10* | *F, 42, urban, -* |
| *P11* | *F, 23, urban, +* |
| *P12* | *H, 34, rural, -* |
| *P13* | *F, 59, rural, +* |
| *P14* | *F, 51, urban, ++* |
