# Supplementary material for: Patients’ Representations of Perceived Distance and Proximity to Telehealth in France: Qualitative Study
Source: J Med Internet Res. 2024 Apr 26;26:e45702. doi: 10.2196/45702 (PMC11087856; doi:10.2196/45702)
Supplement: Multimedia Appendix 2 [file jmir_v26i1e45702_app2.docx]

# Appendix 2: Example of the qualitative analysis process used in this research

| *Transcript* | *Codes* | *Sub-theme* | *Theme* |
| --- | --- | --- | --- |
| “*I found it practical indeed, comfortable*.” (P4) | Convenient | Increase of proximity of access to care | Perceived proximity |
| “*It is so quick, it makes everyday life easier!*” (P8) | Efficient | Functional proximity |  |
| “Now that everything is overbooked in their appointments, (…), we are at about 15 days / 3 weeks for getting any new appointment, both by phone or by Doctolib, in video, it is a little faster” (P13) | Time saving | Decrease of temporal distance from the consultation |  |

*‘(PX)’ stands for the de-identified number assigned to the participant; Doctolib is digital solution used in France for booking appointments with healthcare professionals.* From participants verbatims, we analized the transcripts assigning a code to meanful sentences. Therefore, these codes were grouped into sub-themes and, then, into broader themes.
